# Usage of the HINTS exam and neuroimaging in the assessment of peripheral vertigo in the emergency department

**DOI:** 10.1186/s40463-018-0305-8

**Published:** 2018-09-10

**Authors:** Alexandra E. Quimby, Edmund S. H. Kwok, Daniel Lelli, Peter Johns, Darren Tse

**Affiliations:** 1 Department of Otolaryngology- Head and Neck Surgery, University of Ottawa, S3, 501 Smyth Road, Ottawa, ON K1H 8L6 Canada; 2 Department of Emergency Medicine, University of Ottawa, 501 Smyth Rd, Ottawa, ON K1H 8L6 Canada; 3 Department of Medicine, Division of Neurology, University of Ottawa, 501 Smyth Rd, Ottawa, ON K1H 8L6 Canada; 4 Department of Emergency Medicine, University of Ottawa, 501 Smyth Rd, Ottawa, ON K1H 8L6 Canada; 5 Department of Otolaryngology- Head and Neck Surgery, University of Ottawa, 501 Smyth Rd, Ottawa, ON K1H 8L6 Canada

**Keywords:** HINTS, Head impulse, Neuroimaging, Vertigo, Dizziness

## Abstract

**Background:**

Dizziness is a common presenting symptom in the emergency department (ED). The HINTS exam, a battery of bedside clinical tests, has been shown to have greater sensitivity than neuroimaging in ruling out stroke in patients presenting with acute vertigo. The present study sought to assess practice patterns in the assessment of patients in the ED with peripherally-originating vertigo with respect to utilization of HINTS and neuroimaging.

**Methods:**

A retrospective cohort study was performed using data pertaining to 500 randomly selected ED visits at a tertiary care centre with a final diagnostic code related to peripherally-originating vertigo between January 1, 2010 - December 31, 2014.

**Results:**

A total of 380 patients met inclusion criteria. Of patients presenting to the ED with dizziness and vertigo and a final diagnosis of non-central vertigo, 139 (36.6%) received neuroimaging in the form of CT, CT angiography, or MRI. Of patients who did not undergo neuroimaging, 17 (7.1%) had a bedside HINTS exam performed. Almost half (44%) of documented HINTS interpretations consisted of the ambiguous usage of “HINTS negative” as opposed to the terminology suggested in the literature (“HINTS central” or “HINTS peripheral”).

**Conclusions:**

In this single-centre retrospective review, we have demonstrated that the HINTS exam is under-utilized in the ED as compared to neuroimaging in the assessment of patients with peripheral vertigo. This finding suggests that there is room for improvement in ED physicians’ application and interpretation of the HINTS exam.

## Background

Dizziness is a common presenting symptom in the emergency department (ED), accounting for 2–3% of all US ED visits [1, 2]. In Canada, data from one large tertiary care centre, The Ottawa Hospital (TOH), demonstrated that from 2009 to 2014 dizziness represented almost 2% of all ED visits. Acute vertigo presents a particular challenge to ED physicians, who must differentiate vertigo caused by central nervous system pathology (eg. cerebellar stroke) from that caused by disorders of the peripheral vestibular end organs (eg. vestibular neuritis). Several multicentre studies have cited the prevalence of centrally-originating vertigo among the ED patient population to range between 3.2–12.5% [2–5]. Potential consequences of a missed diagnosis of cerebellar stroke are high, including increased patient mortality [6, 7]. As a result, neuroimaging (computed tomography [CT], and magnetic resonance imaging [MRI] of the brain) is commonly used in the diagnostic work-up of acutely dizzy patients presenting to the ED [8]. In the same data collected from 2009 to 2014, almost 30% of patients presenting to ED with a final diagnosis relating to acute dizziness (a total of 3559 patients) had either CT or MRI of the brain. This neuroimaging cost the hospital an estimated $1.6 million dollars over that period (Le A, Tse D: The cost of dizziness: a cost analysis of overall costs of dizziness at a tertiary care hospital, forthcoming). This figure does not take into account additional costs associated with extended ED stays, or the total amount of cranial ionizing radiation to which patients were exposed.

The HINTS exam was developed as a means of assessing patients with the acute vestibular syndrome (AVS), defined as acute onset and persistent vertigo, gait instability, nausea/ vomiting, nystagmus, and head motion intolerance [9]. This battery of bedside clinical tests consists of three examinations: the head impulse test (HI-), characterization of spontaneous nystagmus (-N-), and test of skew (-TS) [10]. Each of the three components of the HINTS exam is analyzed separately, and a finding in keeping with central vertigo on any one component of the test indicates the need for neuroimaging. The HINTS exam has been shown to have greater sensitivity than neuroimaging in ruling out stroke in patients presenting with AVS, and to outperform other commonly used stroke risk stratification rules [9, 11]. The exam can be performed at the bedside in approximately 1 min and requires no extra equipment or tools.

A recent study demonstrated that despite its ease of use, the head impulse test (HIT) is greatly under-utilized in the ED [12]. Possible reasons for this include a lack of awareness of the test, knowledge of the evidence of its efficacy, and physician confidence in correctly performing or interpreting the exam. Practice patterns in the use of the testing battery of the HINTS exam in the ED have not been previously studied.

The current study sought to assess practice patterns in the assessment of patients with peripherally-originating vertigo presenting to the ED at one large Canadian tertiary care centre. Specifically, we sought to determine relative proportions of HINTS exams and neuroimaging performed on patients presenting with vertigo and dizziness with a final diagnosis of peripheral vertigo, and to better characterize the use of the HINTS exam in examining patients with peripheral vertigo in the ED.

## Methods

### Design

A retrospective cohort study was performed using data obtained over a 5-year period.

### Study objective

The objective of our study was to describe current ED practice patterns in the assessment of acutely vertiginous patients with a final diagnosis of peripheral vertigo. We sought to demonstrate relative proportions of HINTS exams and neuroimaging performed in the assessment of these patients. We also aimed to further characterize ED practice patterns in the use of the HINTS exam, including its interpretation, relative proportions of the exam performed by learners as compared to staff ED physicians, and changes in the use of the HINTS exam over time.

### Setting

The study was performed using data collected prospectively at two campuses of The Ottawa Hospital (TOH), a Canadian academic tertiary care centre. TOH consists of three hospital campuses, two of which have emergency departments. There are > 170,000 ED visits annually at TOH [13].

### Data sources

Patient data was retrieved from The Ottawa Hospital Data Warehouse (TOHDW). TOHDW is a data repository that contains routinely-generated information relevant to all patient visits at TOH. All patient registration, admission, and discharge information, as well as health records data using standardized coding of diagnoses (International Classification of Diseases, 10th revision [ICD-10]) and procedures (Canadian Classification of Interventions) are captured.

### Patient population and data acquisition

We reviewed data pertaining to all patients who presented to TOH ED between January 1, 2010- December 31, 2014 who received one of the following final diagnostic codes (ICD-10): Meniere’s disease (H810), Benign paroxysmal vertigo (H811), Other peripheral vertigo (H813), Dizziness and Giddiness (R42). Data retrieved included patient age, date of presentation, presenting complaint, imaging procedures ordered, referrals made, and final diagnosis codes.

In order to audit the use of the HINTS examination in the emergency department for patients presenting with dizziness, 100 patients were randomly selected who presented each year from 2010-2014, for a total of 500 patients.

Patient unique identifier numbers were retrieved from TOHDW and used to link this sample of 500 patients with patient electronic medical records (EMRs) in order to abstract final charted diagnoses, and whether any bedside test of vertigo (HINTS exam or other) was performed in ED. We also extracted from patient EMRs whether or not the patient was assessed by a trainee (medical student or resident) or a staff ED physician.

Following data extraction from EMRs, we excluded any patients who were captured in our cohort by included ICD-10 codes but whose final charted diagnosis was unrelated to vertigo or dizziness. Examples included diagnoses of pre-syncope, syncope, and other cardiac diagnoses which had been assigned one of our four included ICD-10 diagnostic codes. We also excluded any patients whose EMR indicated that they had left the ED before being assessed by an emergency physician.

### Outcomes of interest

Primary outcomes of interest were: 1) was neuroimaging (CT or MRI of the brain) ordered in the ED, and, 2) was the HINTS exam performed in the ED. Secondary outcomes of interest were: 3) were other bedside tests of vertigo performed (eg. Dix-Hallpike, Romberg, or any single component of the HINTS exam alone), 4) were patients assessed by ED staff physicians or trainees (ie. resident physicians or medical students), and, 5) how were HINTS exam findings charted and interpreted.

When analyzing charted HINTS exam interpretations, we considered the literature citing proper interpretation of the exam. A correct interpretation of the HINTS exam should take into account the results of each of the three components of the test: 1) Head impulse testing: considered abnormal or “positive” when rapid rotation of the head results in loss of fixation of the eyes with a corresponding refixation saccade, which occurs mostly in cases of peripheral vertigo (ie. in vestibular neuritis). In patients with central vertigo, the head impulse test will usually appear to be normal, or “negative”, in that the vestibuloocular reflex remains intact, and the patient’s eyes remain fixed on the target. 2) Examination of patients’ nystagmus: direction-fixed horizontal jerk nystagmus that obeys Alexander’s law, beating away from the affected side, occurs in cases of peripheral vertigo. 3) Vertical skew deviation: absent in cases of peripheral vertigo, with its presence typically indicating a central cause. If any portion of the test is in keeping with a central etiology of vertigo, the HINTS is considered “central”, indicating the need for further investigation (in the form of neuroimaging) (Table 1) [14].

**Table 1:** Interpretation of the HINTS exam

HINTS exam component	Peripheral Vertigo	Central vertigo
Head Impulse Test (HIT)	Loss of eye fixation with head impulse; “positive” or “abnormal”	Intact vestibulo-ocular reflex; “negative” or “normal”
Nystagmus (N)	None or horizontal unidirectional	Vertical, rotatory, or horizontal bidirectional
Test of Skew (TS)	No skew; “negative”	Skew; “positive”

### Data analysis

As our primary and secondary outcomes of interest were descriptive in nature, we performed qualitative data analysis.

### IRB approval

Our study was approved by our hospital REB (Ottawa Health Science Network Research Ethics Board [OHSN-REB], protocol no. 20160726-01H).

## Results

Data pertaining to a total of 10,348 patients who presented to the ED with a final diagnosis related to dizziness or peripheral vertigo between January 1, 2010 and December 31, 2014 was retrieved. Demographic data from this cohort is presented in Table 2. Analyzed encounters were evenly distributed across the 5 years. The majority of discharge diagnoses were represented by the broad ICD-10 diagnostic code of dizziness and giddiness (68%), followed by benign paroxysmal vertigo (15.6%), other peripheral vertigo (14.2%), and Meniere’s disease (1.9%).

**Table 2:** Cohort Demographics

Variable		Total Cohort N (%)	Sample N (%)
Age (Mean)		55	56
Year Presented to ED	2010	1863 (18.0)	100 (20.0)
	2011	1922 (18.6)	100 (20.0)
	2012	2095 (20.2)	100 (20.0)
	2013	2171 (21.1)	100 (20.0)
	2014	2292 (22.1)	100 (20.0)
Presenting Complaint	Dizziness/Vertigo	6314 (61.0)	310 (62.0)
	Syncope/Pre-syncope	1096 (10.6)	50 (10.0)
	General Weakness	544 (5.3)	28 (5.6)
	Nausea and/or vomiting	485 (4.7)	31 (6.2)
	Headache	311 (3.0)	19 (3.8)
	Symptoms of CVA	143 (1.4)	5 (1.0)
	Palpitations/Irregular Heart Rate	105 (1.0)	0 (0)
	Sensory Loss/Paresthesias	35 (0.3)	1 (0.2)
	Gait Disturbance/Ataxia	20 (0.2)	0 (0)
	Other	1295 (12.5)	56 (11.2)
ED Discharge Diagnosis	Dizziness and Giddiness	7036 (68.0)	329 (65.8)
	Benign paroxysmal vertigo	1613 (15.6)	81 (16.2)
	Other Peripheral Vertigo	1473 (14.2)	68 (13.6)
	Meniére’s disease	195 (1.9)	17 (3.4)
Patients who had CT/MRI head		3012(29.1)	139 (27.8)
Total		10,348	500

Analysis of the EMRs of our sample of 500 patients from this cohort revealed that 120 of them (24%) had a final charted diagnosis unrelated to dizziness or vertigo (eg. syncope, pre-syncope) (*n* = 82, 68%), or did not receive a final diagnosis due to having left ED before being assessed by an emergency physician (*n* = 38, 32%). This left 380 patients with a final charted diagnosis related to dizziness or vertigo.

**Fig. 1:**
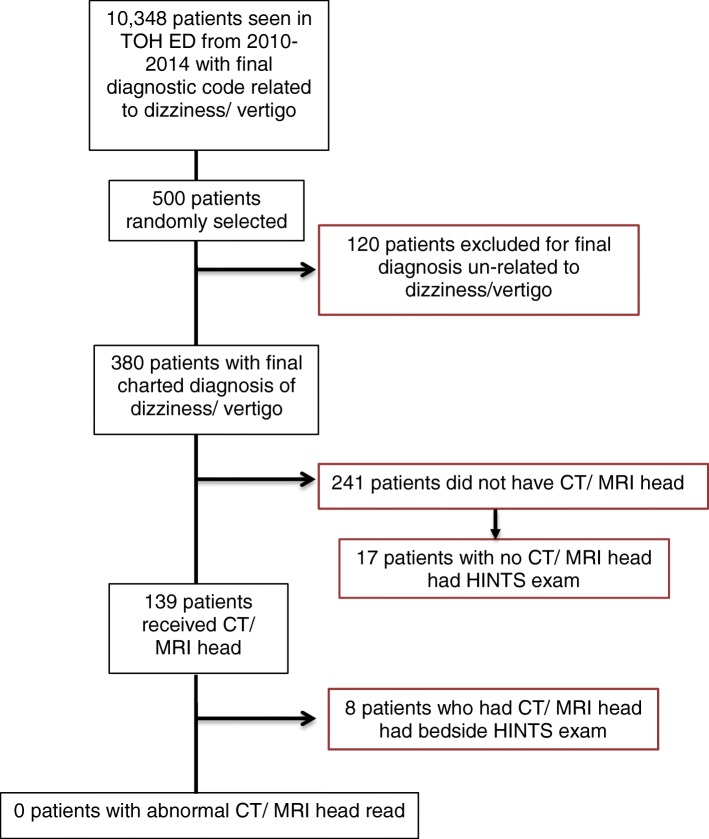
Flow diagram of the use of HINTS and neuroimaging in assessment of patients presenting to ED with dizziness/ vertigo, 2010–2014

Of the 380 patients remaining in our sample who had a final charted diagnosis related to dizziness or vertigo, a total of 139 (36.6%) received neuroimaging in the form of CT, CT angiography (CT-A), or MRI of the head/ brain. Of these, 137 (36%) had non-contrast CT heads, 5 patients (1.3%) had an MRI, and 15 patients (4%) had CT-A. Of these 139 patients who received neuroimaging, 8 (5.8%) had a documented HINTS exam performed at bedside by an ED physician or trainee. Fifty-seven patients (41%) who underwent CT did not have a HINTS exam performed, but did have another bedside test of vertigo performed (any combination of the Romberg test (*n* = 24), the Dix-Hallpike test (*n* = 35), or the head impulse test (HIT) (*n* = 1) or vestibulo-ocular reflex (VOR) testing not otherwise specified (n = 2)). The remainder of patients who had neuroimaging (*n* = 74, 53.2%) did not undergo any bedside testing of vertigo. As anticipated, the total number of reported positive findings on CT/ MRI/CT-A in these patients was 0 (Fig. 1).

Of patients who did not undergo neuroimaging (*n* = 241), 17 (7.1%) had a bedside HINTS exam performed. Ninety-eight patients (40.7%) did not have a HINTS exam performed, but had some other bedside test of vertigo performed (varying combinations of Romberg, *n* = 40; Dix-Hallpike test, *n* = 65; HIT, *n* = 5). The remaining 126 patients (52.3%) who did not undergo CT did not have the HINTS exam or any other bedside test of vertigo performed. Final charted diagnoses of patients who did not undergo neuroimaging or any bedside tests of vertigo included diagnoses such as “dizziness NYD”, “vertigo NYD”, “benign vertigo”, “peripheral vertigo”, “Meniere’s”, and “BPPV”.

**Fig. 2:**
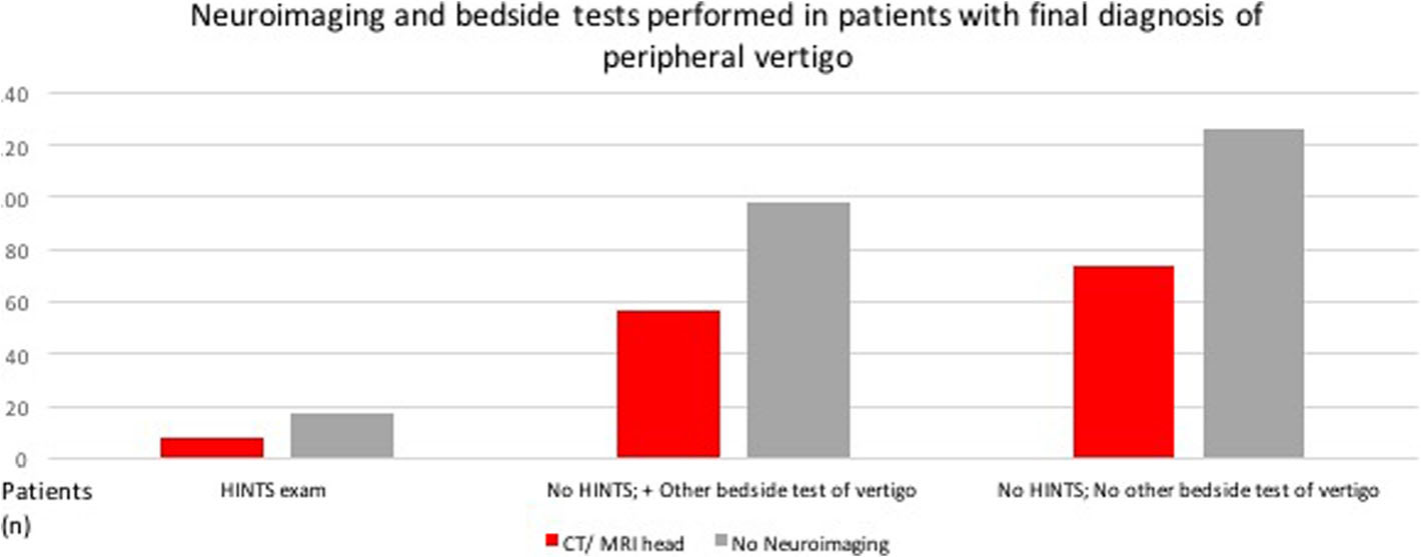
Relative proportions of patients receiving neuroimaging, HINTS exams, and other bedside tests of vertigo

Relative proportions of patients receiving neuroimaging, HINTS exams, and other bedside tests of vertigo are displayed in Fig. 2.

**Fig. 3:**
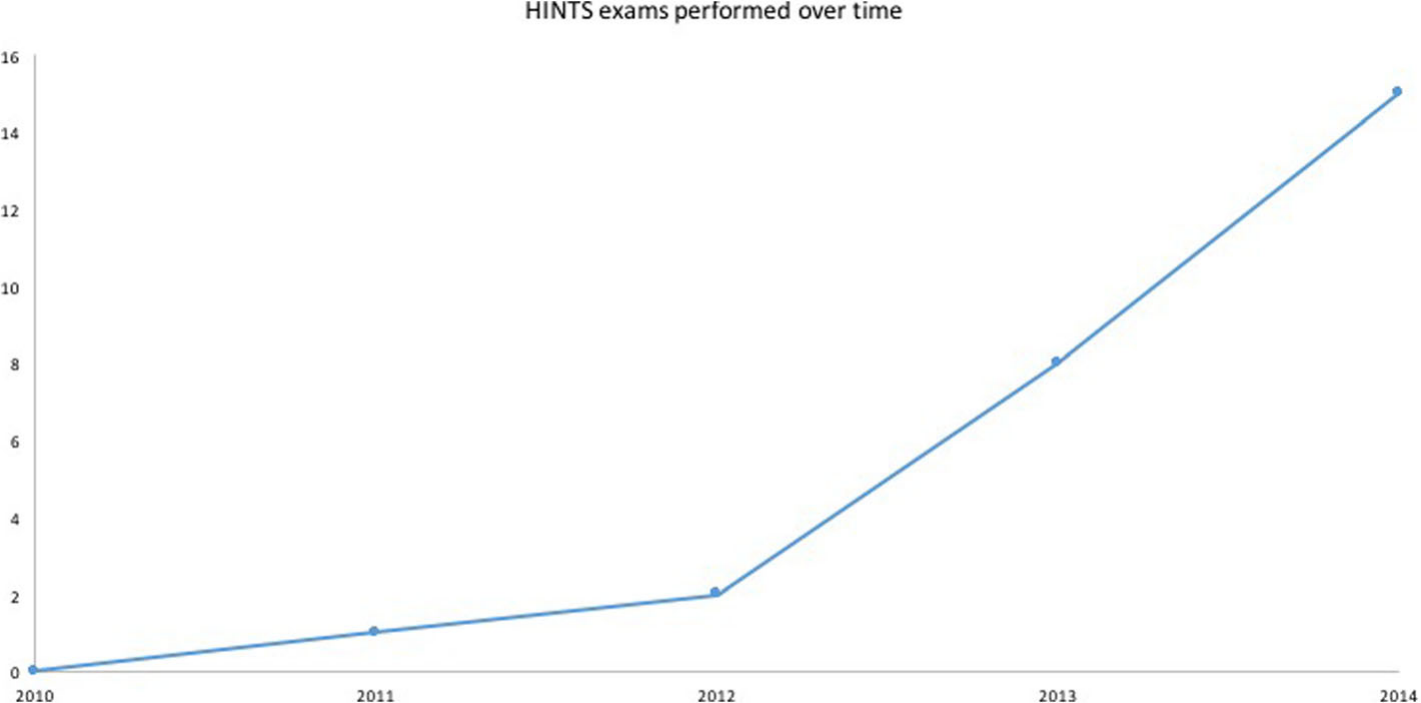
Number of HINTS exams performed by year, 2010–2014

Analysis of the number of HINTS exams performed each year over our 5-year study period revealed a trend of increasing numbers of HINTS exams over time, which was statistically significant (*p* < 0.005) (Fig. 3). In particular, an increase in the number of HINTS exams was noted between the years 2012 and 2013. Dr. Newman-Toker, the originator of the HINTS exam, presented at Emergency Department rounds at TOH on the utility of the HINTS exam in October 2012.

Of the 26 HINTS exams performed on patients in our sample, 7 (27%) of these were performed by trainees – either resident physicians or medical students – and 19 (73%) by staff ED physicians.

Analysis of the charted interpretations of the HINTS exams performed in our sample revealed that almost half of the time, the exam was charted as “HINTS negative” (*n* = 11, 44%). Of these 11 “HINTS negative” patients, 4 (36.3%) underwent CT. For patients on whom the exam was charted with the results of its three separate components, neuroimaging was appropriately ordered in 50% of the cases. There were 11 patients (44%) for whom charted interpretations of the HINTS examination would suggest HINTS central (ie. any one component of the exam positive OR no nystagmus present), indicating the need for neuroimaging. Of these 11 patients, neuroimaging was subsequently ordered in 4 (36.3%) (Table 3).

**Table 3:** Charted ED interpretations of HINTS exams, and neuroimaging ordered

Charted Interpretation of HINTS exam		Charted Interpretation corresponds to HINTS Central, or HINTS Peripheral?	Neuroimaging Indicated?	Patients with charted interpretation (n)	CT performed? (n)	CT positive findings?
“HINTS negative” (n = 11)		NA	NA	11	Yes (4)	No
Results of each of 3 components of exam charted (n = 14)	HIT +ve, Nystagmus right, Test of skew –ve	Peripheral	No	1	No (0)	\
	HIT normal, nystagmus none, test of skew normal	Central	Yes	3	Yes (2)	No
	HI = + lag, Nystagmus = N, TS = N	Peripheral	No	1	No (0)	\
	HIT abnormal, Nystagmus horizontal, Test of skew normal	Peripheral	No	1	No (0)	\
	No lag on head impulse, unilateral nystagmus, lateral skew	Central	Yes	1	Yes (1)	No
	Head impulse abnormal, Nystagmus vertical, no skew	Central	Yes	1	Yes (1)	No
	HINTS = peripheral cause	Peripheral	No	1	No (0)	\
	Symptoms reproducible with quick head turn to the right, no nystagmus, no skew	Peripheral	No	1	No (0)	\
	Head impulse abnormal, Nystagmus bilateral, Test of skew positive	Central	Yes	1	No (0)	\
	HIT –ve, N –ve, TS –ve	Central	Yes	1	No (0)	\
	Head impulse +, no skew, no nystagmus	Peripheral	No	1	No (0)	\
	HI + ve, N –ve, T –ve	Peripheral	No	1	No (0)	\

## Discussion

The HINTS exam is a well-validated tool to rule out stroke in patients presenting with AVS, with greater sensitivity than neuroimaging [9, 11]. However, the exam has previously been shown to be under-utilized in the ED [12]. Our study expands upon the existing literature by qualitatively characterizing practice patterns in the use of the HINTS exam and neuroimaging for the assessment of patients with peripheral vertigo in the ED.

We have demonstrated that of patients presenting to ED with dizziness or vertigo and a final diagnosis of peripheral vertigo, a high proportion (36%) undergo neuroimaging, and the HINTS examination is relatively under-utilized in this population in comparison (7%). Furthermore, when the HINTS exam was used in our ED cohort, it was often applied inappropriately, with neuroimaging still ordered in cases of HINTS peripheral.

Our demonstration of a low overall proportion of HINTS exams performed on vertiginous patients in whom neuroimaging was ordered may be a result of several factors. First, awareness of the HINTS exam may be low among ED physicians. This is supported by our demonstration that use of the HINTS exam at our centre increased over time, especially since Dr. Newman-Toker’s presentation of the examination in 2012. Similarly, publications citing evidence for the efficacy of the HINTS exam only first appeared 2009, and have increased in number since.

Second, ED physicians and trainees may be uncomfortable or unfamiliar with the HINTS exam technique and interpretation. In the published literature, the accuracy of the HINTS exam has been demonstrated in the setting of being performed by specialist physicians, including neurologists, neuro-ophthalmologists, and expert-trained emergency room physicians [14]. This may lead ED physicians to avoid the exam altogether, or not trust the reliability of the results they obtain. In our sample, almost 50% of patients who underwent the HINTS exam had their results charted as “HINTS negative”. Of these patients, approximately one third (36%) underwent subsequent neuroimaging, with the remainder receiving no further testing. As well, of patients on whom ED charting of HINTS exam results corresponded to HINTS central (*n* = 11 or 44% of patients on whom the exam was performed), indicating the need for subsequent neuroimaging, only 36% went on to have CT or MRI. In many of these cases, even though the HINTS exam was fully documented, it was inappropriately interpreted as peripheral in 28.5% of patients who had no nystagmus present. These findings suggest ambiguity in ED physicians’ interpretation of the HINTS exam. Extensive use of neuroimaging may stem from practitioners’ lack of self-confidence in their technique and interpretation of the exam. It is possible that with adequate training on technique and interpretation of the exam, the number of HINTS exams performed and properly interpreted in the ED would increase, and the use of neuroimaging would decrease. Our demonstration of a relatively larger proportion of HINTS exams being performed by staff ED physicians (73%) as compared to residents and medical students (27%) also suggests the possibility for improvement in use of the exam with teaching targeted to various levels of medical training.

Possible implications of broadening usage of the HINTS exam in the ED are improved patient care outcomes, decreased ED wait-times for neuroimaging, and decreased exposure to ionizing radiation. Improved patient satisfaction is likely to result. Decreasing the use of neuroimaging will also result in healthcare cost savings.

Our study expands upon existing literature demonstrating HINTS under-utilization in the ED. We have quantitatively demonstrated proportions of HINTS exams and neuroimaging performed in the ED assessment of patients with peripheral vertigo, as well as qualitatively shed light on these practices. Though an overall small number of HINTS exams were performed, we have qualitatively demonstrated charted ED interpretations of these HINTS exam, relative proportions of the exam performed by trainees as compared to staff physicians, and changes in practice patterns over time. By depicting the impact of teaching on the HINTS exam– that is, how HINTS ED usage increased at our centre following demonstration of the exam by Newman-Toker – we have shown that these practice patterns are amenable to change with appropriate educational initiatives.

Several important factors must be taken into consideration when interpreting our results. The HINTS examination should only be used in patients with AVS: that is, acute onset and persistent vertigo, gait instability, nausea/ vomiting, nystagmus, and head motion intolerance. Of our sample of 380 patients with coded diagnoses of dizziness or vertigo, it is likely that a proportion of these did not meet the criteria for AVS, making the use of a HINTS examination inappropriate. Indeed, in some patients in our cohort with a final charted diagnosis of BPPV, a HINTS exam and neuroimaging were still performed. It is difficult to ascertain which patients truly met criteria for AVS on the basis of charted ED notes. Therefore, the calculation of 7% (*n* = 25) of patients with dizziness/ vertigo who underwent a HINTS exam is likely an under-estimate of the percentage of patients who appropriately underwent HINTS testing. However, of 139 vertiginous patients who underwent neuroimaging (CT or MRI head), only 6% of these (*n* = 8) underwent a HINTS exam in advance, indicating the HINTS exam is under-utilized in patients in whom ED physicians are concerned about central vertigo. Other limitations of our study include its retrospective design and our reliance on charted ED diagnoses. Data used in our sample was retrieved on the basis of ICD-10 diagnostic codes, which are dependent on both accurate charting by physicians and coding of charted diagnoses by non-clinical clerks. We also cannot comment on the sensitivity nor specificity of the HINTS exam when used by ED physicians, since we only analyzed patients with a final diagnosis of peripheral vertigo. However, by analyzing trends in charted interpretations of the HINTS exam performed by ED physicians, we were able to show that ambiguity regarding the proper interpretation of the test exists in this population, and that there is therefore room for improvement with additional training of the proper technique and interpretation of the HINTS exam.

Given the findings of this qualitative analysis, future directions include implementing an educational campaign within our centre’s ED with the goals of increasing awareness, proper technique, documentation, and interpretation of the HINTS exam. By doing this, we would hope to institute a measurable change in practice, with increased use of the HINTS examination and decreased use of neuroimaging in the assessment of acutely vertiginous patients in the ED. If use of the HINTS exam in the ED could be increased and a more robust dataset curated, this information could be used to develop an algorithm for the assessment of acutely dizzy patients in the ED. Though a single centre study, we believe that the observed trends in the use of neuroimaging in our patient population are likely consistent with ED practices more generally, as is suggested by concordance of our results with other studies [12, 15]. Through implementing measurable change in the utilization of the HINTS exam at our own centre, our hope would be to confer a generalizable shift in practice patterns.

## Conclusion

Dizziness is a common presenting complaint in the emergency department, and can be challenging for ED physicians who must exclude potentially life-threatening central causes of vertigo. The HINTS examination, designed to differentiate peripheral and central vertigo, is significantly under-utilized compared to neuroimaging in the ED assessment of patients with peripheral vertigo. We have demonstrated that there is room for improvement in the application and interpretation of the HINTS exam in the ED, creating the potential for both improved patient care outcomes and healthcare cost-savings.
